# The distress context of social calls evokes a fear response in the bat *Pipistrellus abramus*

**DOI:** 10.1242/jeb.246271

**Published:** 2023-11-28

**Authors:** Kazuki Yoshino-Hashizawa, Yuna Nishiuchi, Midori Hiragochi, Motoki Kihara, Kohta I. Kobayasi, Shizuko Hiryu

**Affiliations:** Graduate School of Life and Medical Sciences, Doshisha University, 1-3 Tatara-Miyakodani, Kyotanabe, Kyoto 610-0394, Japan

**Keywords:** Distress call, Electrocardiogram, Heart rate, Freezing response, Auditory oddball paradigm

## Abstract

Bats primarily use sound information, including echolocation, for social communication. Bats under stressful conditions, for example when confronted by a predator, will emit aggressive social calls. The presentation of aggressive social calls, including distress calls (DCs), is known to increase heart rate (*f*_H_), but how this change in *f*_H_ is related to the bat's sound perception and how this evokes behaviors such as the fear response is unknown. Herein, we show that the perception of a distress context induces freezing behavior as a fear response in bats. We found that bats responded by freezing and displayed increased *f*_H_ when they were presented with a conspecific donor bat in a distress situation evoked by gentle poking with a cotton swab. In addition, when we presented two types of auditory oddball paradigms with different probabilities of DCs and echolocation calls (ECs), the bats' *f*_H_ increased when DCs were presented as deviant or control stimuli within standard ECs but did not increase when DCs were presented as standard stimuli. These results suggest that the situational context created by the frequency of sound presentation, rather than simply a single sound feature, induces *f*_H_ increases and freezing as fear responses in bats.

## Teaser

**Summary:** The electrocardiograms of captive *Pipistrellus abramus* show that their heart rate increases as a fear response when the bats hear sounds with a distress context.

## INTRODUCTION

Echolocating bats rely heavily on sound information as both a means of learning about their external environment and a factor in determining their own behavior. Bats are social animals that communicate primarily through sound (Chaverri et al., 2018). The social calls of echolocating bats include aggressive calls, warning calls, mating calls, songs and isolation calls; which one is used depends on the context (Bohn et al., 2008; Davidson and Wilkinson, 2004; Fenton, 2003; Gelfand and McCracken, 1986; Pfalzer and Kusch, 2003). For example, distress calls (DCs), a form of social call, are produced by many animals when they are in situations of extreme danger such as being caught by a predator or tangled in a catch net (Carter et al., 2015; Conover, 1994; Lingle et al., 2012; Ruiz-Monachesi and Labra, 2020), and bats express DCs when in a stressful or dangerous situation (Chaverri et al., 2018; Fenton et al., 1976). Previous studies have reported the possible roles of the DCs of bats as direct responses to predators, as warnings to kin or non-kin individuals, and as a means of attracting conspecifics or heterospecifics for mobbing predators (Arnold et al., 2022; August, 1985; Carter et al., 2015; Knörnschild and Tschapka, 2012; Russ et al., 2004; Ryan et al., 1985). In addition, the acoustic structures of such vocalizations may contain information about the caller, such as body size, health status and fear status (August and Anderson, 1987; Gadziola et al., 2012; Hechavarría et al., 2020; Jiang et al., 2017). Besides the acoustic analysis of these social calls and research concerning their functions, it is also important to study changes in the internal states of the bats receiving social calls. For example, it has been reported that DCs evoke changes in neurotransmitters and stress hormones in the amygdala as a fear-related response in bats (Mariappan et al., 2013). Heart rate (*f*_H_) is an important parameter in the evaluation of internal states, as it can indicate tense, aggressive or appeasement states evoked via the autonomic nervous system. A previous study reported that the magnitude and duration of elevated *f*_H_ were correlated with the level of evoked aggression in emitter bats (Gadziola et al., 2012). Some studies have used changes in *f*_H_ to detect the fear level in social calls, and these studies have reported that aggressive stimuli evoked *f*_H_ increases as a fear response in receiver bats (Gadziola et al., 2016; Hechavarría et al., 2020; Ma et al., 2010). Thus, the usefulness of *f*_H_ changes for assessing internal states, such as emotions, has been demonstrated in studies of bats focusing on the recipients of social calls. The presentation of aggressive social calls, such as DCs, is known to increase the receiver bats' *f*_H_, but how the *f*_H_ change is based on the bat's perception and how this leads to a given behavior (i.e. a fear response) have yet to be investigated. Evaluating both behavior as an external state and physiological changes as an internal state is important because internal states profoundly alter perceptions and behavior. Based on this, we hypothesize that the communicative sound-based context changes the receiver's internal state, which, in turn, changes the receiver's behavior. Recent studies have shown that contexts consisting of several vocalization sequences, rather than a single sound stimulus, may be important for bat communication (Amit and Yovel, 2023). Acoustic communication and the context (mentioned above) change the internal state of a receiver. Thus, we expected that, instead of a single DC stimulation, the recognition of a distress context or situation would increase *f*_H_ as a fear response. In this study, therefore, we investigated whether (1) fear responses (freezing) and *f*_H_ increases were observed in subject bats when confronted with the stimulus of a donor bat in distress emitting a DC and (2) the acoustic context of the DCs evoked a fear response using the auditory oddball paradigm, which consists of different presentation probabilities of DCs. We examined the fear response and cognitive processes in a distress context through these experiments.

## MATERIALS AND METHODS

### Study species and ethical status

The study species was the Japanese house bat *Pipistrellus abramus* (Temminck 1840), an insectivore and aerial hunter in the order Vespertilionidae. For echolocation, this species uses frequency-modulated (FM) pulses that sweep downward from initial frequencies around 80 kHz to terminal frequencies (TF) around 40 kHz (as recorded in the field: Fujioka et al., 2011; Hiryu et al., 2008). Auditory sensitivity exhibits a broad U-shape ranging from approximately 4 to 80 kHz and a low threshold between 20 and 50 kHz, with the highest sensitivity in the 35–50 kHz frequency range (Boku et al., 2015; Goto et al., 2010).

Subjects were captured from a colony roosting near the campus of Doshisha University in Kyotanabe, Japan, or were birthed in our laboratory from captive pregnant females. The bats were kept in plastic cages (25 cm×17 cm×17 cm) and allowed free access to mealworms and water. Eighteen adult bats (8 males and 10 females) were used in this experiment. All experiments complied with the Principles of Animal Care [publication no. 86-23 (revised 1985) of the National Institutes of Health] and all Japanese laws. All procedures were approved by the Animal Experiment Committee at Doshisha University.

In this study, three different experiments were conducted (Fig. S1). Each experimental procedure is described below.

### Experiment I: behavioral response to a donor bat stimulus

We recorded sounds in the experimental environment and the behavioral responses of subject bats when confronted with a donor bat in a state of distress. Seven bats (4 females and 3 males) were used as the subject bats in this experiment. The experiment was carried out in a cylindrical arena (70 cm in diameter, 50 cm in height) surrounded by a metal net wall in a soundproof chamber (1.4 m×1.4 m×2.1 m) with red light [G-82H(R), ELPA, Osaka, Japan] that prevented the animals from relying on visual information (Hope and Bhatnagar, 1979). A video camera (DMK33UX273, The Imaging Source, Bremen, Germany, frame rate: 10 frames s^−1^) was set 70 cm above the center of the arena to observe the entire area. Acoustic signals were recorded with a microphone (Anabat SD2 CF Bat Detector, Titley Scientific, Brendale, QLD, Australia) placed on the arena floor. The sound recordings were conducted through a data acquisition system (BNC-2110/PXIe-6358/PXIe-1073, National Instruments, Austin, TX, USA) and a custom-written program in Labview 2016 (National Instruments, sampling rate: 500 kHz/channel).

Three adult male bats were used as stimulus donors, all of which were kept in separate rearing cages from the time of capture. A stimulus donor bat was placed in a metal grid cage (17 cm×17 cm×17 cm) with an open top. We set three conditions of stimuli in this experiment: distressed bat (DB), non-distressed bat (NDB) and control (no bat in the chamber) conditions. In the DB condition, the experimenter gently poked the donor bat with a cotton swab to evoke a DC as in previous studies (Gadziola et al., 2012). For the NDB condition, the donor bat was able to move freely within the cage without a stress stimulus. For the control, only the cage (without a donor bat) was present in the arena.

To prevent echolocation until the recording began, the experimenter gently placed a subject bat covered with a plastic cup (5 cm in diameter and 3 cm high) at the starting point, 30 cm away from the center of the arena. Then, the cage with the stimulus donor bat (no bat in the control condition) for each condition was placed in the arena about 20 cm away from the subject bat. As soon as recording began, a rope attached to the top of the cup was pulled to release the cover over the subject bat. Each condition was presented for 3 min (one trial) and one or two trials were conducted for each of the three conditions for each individual (DB: 10 trials from a total of six bats, NDB: 10 trials from a total of six bats, control: 11 trials from a total of seven bats; Table S1). A small number of trials was chosen to exclude the effect of habituation to the stimuli.

The response behaviors of the subject bats were classified as ‘fly’, ‘crawl’ or ‘stay’. The reaction time from the start of the stimulus (the time when the cup over the subject bat was removed) until the subject flew or crawled was also recorded. Sound data were analyzed by a custom-written program in MATLAB (R2019b, MathWorks, Natick, MA, USA). One hundred calls that were randomly selected from recorded calls 6 dB peak to peak (p–p) greater than the noise level were manually classified as echolocation calls (ECs) or distress calls (DCs). Note that the DCs were emitted only by the donor bat, while the ECs from the donor bat were not distinguished from those of the subject bat. The DCs were further classified into three types as follows: DCFM (down FM pattern with lower TF than echolocation calls and a duration shorter than 10 ms), DCNB (noise burst lasting more than 10 ms), or DCO (other calls that were not classified as either DCFM or DCNB). The initial frequency (IF) and the TF of DCFM were analyzed based on a spectrogram (2048 point windows, −25 dB threshold from the peak power) to check the distribution as the stimuli for experiment III.

### Experiment II: ECG measurement for behavioral stimuli

In this experiment, we measured the electrocardiogram (ECG) as a physiological parameter to examine the internal states of the subject bats in real time via autonomic nervous system functions. Five adult bats (2 females and 3 males) were used as subjects in this experiment. Subject bats were anesthetized with 2–4% isoflurane (Pfizer, New York, NY, USA) during surgery, and 2% xylocaine jelly (Sandoz, Rotkreuz, Switzerland) was applied to the surface of the skin after the scalp fur was shaved. Then, the skin and muscles over the cranium were retracted. The ECG wires were directed to the electrode socket and fixed with dental cement (Super-Bond C&B, Sun Medical Company, Moriyama-shi, Shiga, Japan/UNIFAST III, GC, Tokyo, Japan) on the cranium (see Boku et al., 2015; Furuyama et al., 2018). The ECG electrodes (silver wires) were implanted in the subject bat in three different positions (Lead II) as follows: left leg (positive), right thumb (negative) and right leg (reference), and fixed under the skin. Lead II is one of the highest signal-to-noise ratio (SNR) measurements for ECG in echolocating bats (Mihova and Hechavarría, 2016).

This experiment was carried out in the same soundproof chamber using the same behavioral recording system as in experiment I. Subject bats participated in the experiment after at least 1 week of recovery following surgery. On the day of the experiment, the subject bat was immobilized with a soft sponge and placed on the arena floor approximately 20 cm away from the stimulus donor cage. The electrode socket of the subject was connected to an ECG measurement system (C3410/C3314/C3216/C3100, Intan Technologies, Los Angeles, CA, USA). Before the recording, we tested whether the electric impedance of all ECG electrodes was <ca. 25 kΩ before the measurement to check that the electrodes were functioning. The ECG was recorded by RHX software (Intan Technologies) using 30 kHz sampling and was synchronized to video recordings by a pull-down trigger signal.

Five adult bats (3 females and 2 males) were used as stimulus donors. Four of the donor bats were kept in separate rearing cages from the time of capture of the subject bats. One subject–donor pair was kept in the same cage from the time of capture. All pairs were of the same sex to avoid sexual interaction (the details are listed in Table S2). We set the DB and NDB conditions the same as in experiment I, but two control conditions were set: one in which only the cage without a stimulus donor bat was presented (cage control condition) and the other in which no cage was presented (no-cage control condition). These four conditions were presented randomly, and each condition was presented to the subject bat for at least 1 min (one trial). The ECG recordings were stopped if the signal-to-noise ECG condition of the subject was poor or the donor bats appeared fatigued, so that the recording time ranged from 8 to 21 min.

### Experiment III: ECG measurement for acoustic stimuli

To avoid confounding stimuli, such as odor from the donor bat or movements of the experimenter, and to measure responses based on acoustic information, the ECG was recorded while pre-recorded acoustic stimuli (calls emitted by a donor bat) were presented from a loudspeaker instead of by a donor bat. The acoustic parameters of the DC stimuli in this experiment were within the normal range of the DCFM in experiment I.

The experiment was carried out in the same soundproof chamber as in experiment I, and the surgical procedure was the same as in experiment II. The loudspeaker (PT-R7III, Pioneer, Tokyo, Japan) was installed on the floor of the arena in the chamber and connected to an amplifier (STR-DH190, Sony, Tokyo, Japan) and D/A converted via an audio interface (Rubix44, Roland, Hamamatsu, Shizuoka, Japan, sampling rate: 192 kHz). The subject bat was immobilized with a soft sponge and placed on the floor of the arena, approximately 10 cm away from the loudspeaker. The stimulus calls were pre-recorded using a sound acquisition system (CM16/CMPA/UltraSoundGate 116Hme, Avisoft-Bioacoustics, Glienicke/Nordbahn, Germany) from a male donor bat while the experimenter held the bat in their hand, maintaining a constant distance between the microphone and the bat, to elicit ECs and DCs as in previous studies (Carter et al., 2015; Hörmann et al., 2021; Jiang et al., 2017; Russ et al., 2004). Each recorded sound sequence was trimmed into a 3 s echolocation call period and a 1.5 s distress call period. We selected the DCFM type as the distress stimulus in this experiment because a previous study reported that the DCFM type increased *f*_H_ by more than the DCNB type (Gadziola et al., 2016). Then, successive vocalizations were normalized for amplitude and adjusted to a length of 3 s by repeating the distress call word cutoff at 1.5 s twice. In this experiment, the amplitude level of the stimulus was adjusted such that the sound pressure reaching the bat, which was placed approximately 10 cm from the center of the loudspeaker, was 100 dB p–p sound pressure level (SPL). Although the SPL of the stimuli should be controlled by the loudness of the bats, it is difficult to control the loudness of the sound stimuli because of the different pulse duration and the frequency range of the EC and DC sound stimuli used in this study, so we controlled the stimulus level by the p–p value of the stimulus sound. We confirmed from our previous experiment that a SPL of 100 dB p–p at 10 cm from the loudspeaker was sufficient to elicit an auditory response in *P. abramus* (Boku et al., 2015). A microphone measurement system (5935/4939-A-011, Brüel & Kjær, Virum, Denmark) and an oscilloscope (TBS2104, Tektronix, Beaverton, OR, USA) were used for this adjustment.

Here, we adopted the flip-flop auditory oddball paradigm (Näätänen et al., 1978; Ulanovsky et al., 2003) to examine changes in *f*_H_ induced by the acoustic stimuli rather than unexpectedness. DCs and ECs were used as stimuli in this experiment. The oddball session comprised 90 standard stimuli and 10 deviant stimuli that occurred in a random series (see Fig. S2). The interstimulus interval was set at 30 s to allow the *f*_H_ to return to a stable level before stimulus presentation, referencing a *f*_H_ study for an acoustic task in birds (Ikebuchi et al., 2003). The control session consisted of 10 deviant stimuli with an interstimulus interval of 30 s. Each trial consisted of one oddball and one control session (90 standard, 10 deviant and 10 control stimuli). Note that the number of presentations of the standard stimuli was increased as a result of the forced insertion of the standard stimuli after the deviant stimuli. We set two types of oddball paradigms by flipping the standard and deviant stimuli. We conducted 11 trials of the EC-standard oddball paradigm (EC sounds are standard stimuli and DC sounds are deviant stimuli) using six bats (4 females and 2 males; each subject made between one and five trials) and five trials of the DC-standard oddball paradigm (DC sounds are standard stimuli and EC sounds are deviant stimuli) using four bats (2 females and 2 males from the default oddball paradigm participated; each subject made one or two trials). For each bat, the measurements lasted approximately 1 h.

### ECG signal analysis

ECG signal processing was conducted using a custom-written MATLAB script (R2019b, MathWorks). The ECG was obtained from the potential difference of two electrodes between the positive and negative terminals attached to the subjects. The ECG was bandpass filtered (40 to 150 Hz) to detect the peak of the R waves and to eliminate outliers based on the amplitude distribution (a range of 5% to 95% was retained). The instantaneous *f*_H_ was calculated from the inverse of the valid R wave interval of the ECG. To analyze the effect of the stimuli, the *f*_H_ was binned by 1 s before/after the onset of the stimuli via averaging. The binned *f*_H_ was analyzed using a −10 to 90 s period in experiment II (see below) and a −10 to 30 s period in experiment III (see below) that was fitted to each interstimulus interval (where the stimulus onset was set to 0 s). Finally, the *f*_H_ was normalized for the average of each −10 to 0 s period for the onset per trial (in experiment II) or per stimulus (in experiment III) to evaluate values that considered individuality and temporary activeness and thus the relative effect of the stimuli.

### Statistical analysis

All statistical analyses were performed using a custom-written MATLAB script (R2023a, MathWorks). To test for significant deviation from a normal distribution, we used the MATLAB ‘lillietest’ function. Based on the results, we used *post hoc* non-parametric tests comparing average ranks (MATLAB functions ‘kruskalwallis’ and ‘multcompare’, with Bonferroni correction) for the relative *f*_H_ bins after the stimulation period with the stimulation group in experiments II and III. In our study, we used only non-parametric tests because all data deviated from a normal distribution.

## RESULTS

### Experiment I: behavioral response to stimulus donor bats

**Fig. 1.**
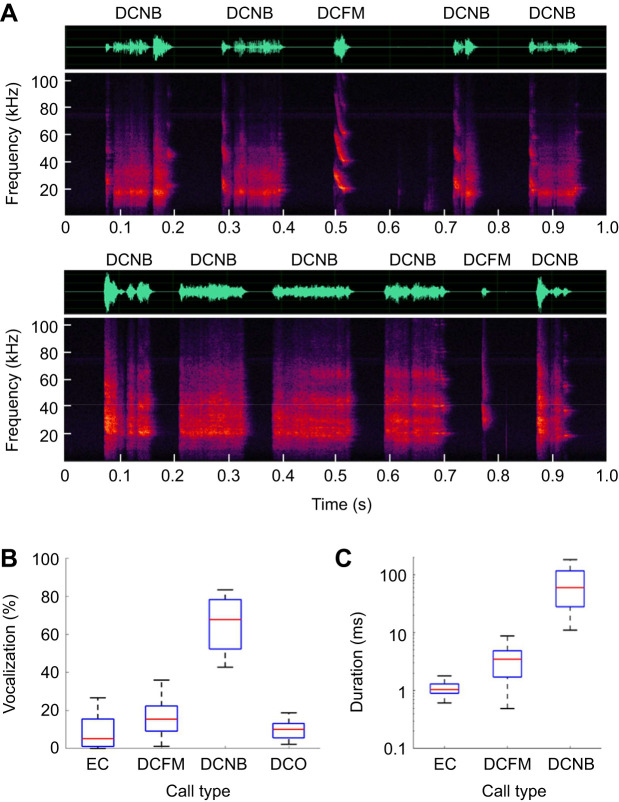
**Vocalizations of the donor *Pipistrellus abramus* in the distress situation.** (A) Example of a series of distress vocalizations (DCNB, distress call noise burst; DCFM, distress call frequency modulated) from two different individuals. Each panel shows a time waveform (top) and a spectrogram (bottom). (B) Box plots of vocalization rate versus call type (EC, echolocation call; DCFM; DCNB; DCO, distress call other). (C) Box plots of duration versus call type. Here, DCO was eliminated because of a lack of classification rules. Each box plot shows the median (red line), quartiles (bottom and top edges of the boxes), and the minimum and maximum range (whiskers) in 10 trials of the distressed bat (DB) condition (total of 11,187 calls from a total of three male donor bats).

In 10 trials using the DB condition, a total of 11,187 calls were recorded from three male donor bats. Fig. 1A shows example vocalization sequences of two donor bats; the vocalizations have complex temporal patterns, including DCFM and DCNB types. In all 10 trials with the DB condition, DCNB had the highest vocalization rate (Fig. 1B). The average duration of EC in *P. abramus* was 1.12±0.33 ms (mean±s.d.), whereas the duration of DCNB was the longest (71.77±45.01 ms), at times exceeding 100 ms (Fig. 1C).

**Fig. 2.**
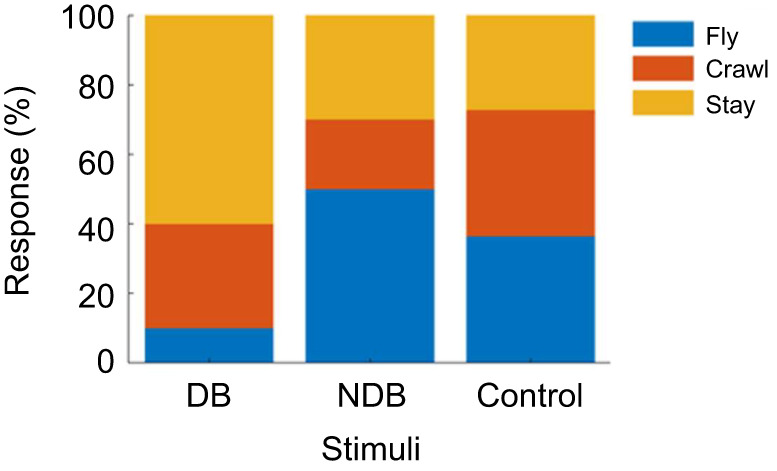
**Distribution of behavioral responses in experiment I.** The distressed bat (DB) and non-distressed bat (NDB) stimuli each show 10 responses from a total of six subject bats, respectively, and the control shows 11 responses from a total of seven subject bats.

Fig. 2 shows the percentages of the behavioral responses of the subject bats in each condition (the result of each trial is shown in detail in Table S1). In the DB condition, subjects were most likely to respond with ‘stay’ (60.0%) and least likely to respond with ‘fly’ (Fig. 2). In the NDB condition, in contrast, ‘fly’ was the dominant response at 50.0%. In the control condition, the percentage of ‘stay’ was 27.3% and those of ‘crawl’ and ‘fly’ were both 36.4%. The reaction times for the ‘fly’ or ‘crawl’ response were 95.25±49.72 s (mean±s.d.) in the DB condition, 97.29±51.78 s in the NDB condition, and 64.13±44.85 s in the control condition.

### Experiment II: ECG measurement for behavioral stimuli

**Fig. 3.**
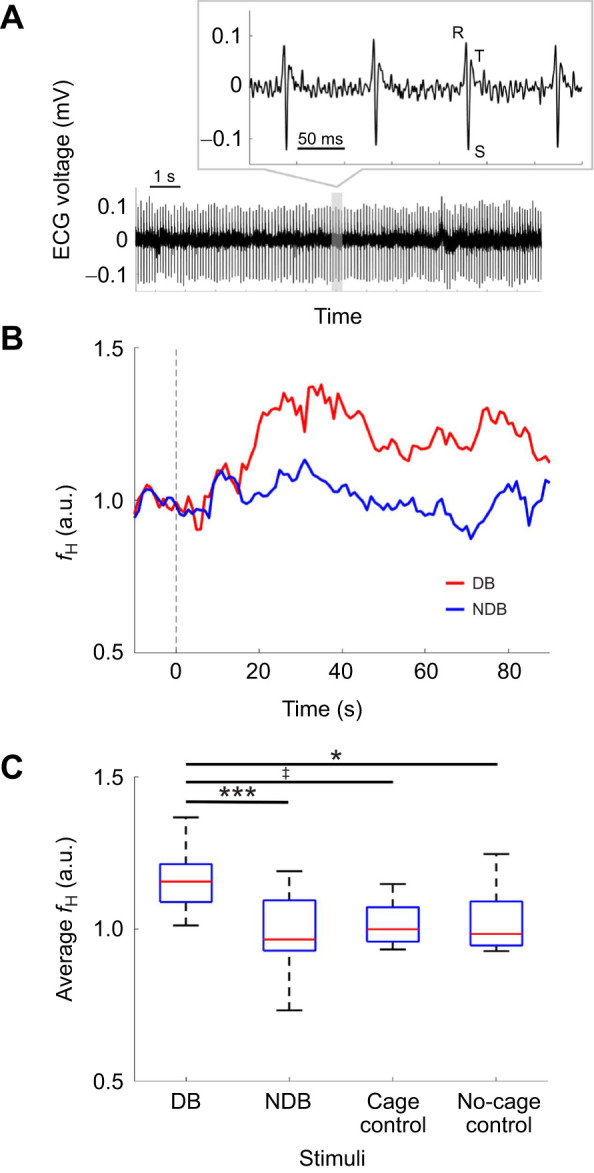
**Results of ECG analysis in experiment II.** (A) Electrocardiogram (ECG) recorded with 40–150 Hz bandpass filter. The R, S and T waves are clearly visible in each cardiac beat from a subject bat. (B) Example of averaged relative heart rate (*f*_H_) (a.u., arbitrary units) responses to the DB and NDB stimuli in one subject. The vertical gray dotted line at 0 s shows the onset of stimulation. (C) Distribution of average relative *f*_H_ from 0 to 90 s after the onset of stimulation. Each box plot shows the median (red line), quartiles (the bottom and top edges of the boxes), and the minimum and maximum range (whiskers). Statistical significance is indicated by the symbols: ^‡^*P*<0.1, *P<0.05, ***P<0.001 (multiple comparison test with Bonferroni correction; total of 60 trials in four conditions from a total of five subject bats).

For each of the four conditions (DB, NDB, cage control and no-cage control), five subject bats made between zero and four trials each (DB: 15 trials, NDB: 21 trials, cage control: 9 trials, no-cage control: 15 trials; see Table S2 for details, including information on donor–subject bat combinations). Recorded ECGs had stable SNRs with no clips (approximately ±0.1 mV range) in all trials, as shown in Fig. 3A. The ECGs included clear R, S and T waves, as in other species (Currie, 2018). The *f*_H_ ranged from approximately 200 to 600 beats min^−1^ through all measurements. Fig. 3B shows an example comparing the change in relative *f*_H_ during 90 s after stimulus presentation in one individual in the DB and NDB conditions. The relative *f*_H_ increased by a maximum of ∼40% after the onset of the DB condition despite being maintained after the onset of the NDB condition. The averaged *f*_H_ from 0 to 90 s after stimulus onset was significantly different among the three conditions (Kruskal–Wallis test, *P*<0.001). Furthermore, *f*_H_ in the DB condition was significantly higher than that in the NDB condition (multiple comparison test with Bonferroni correction, *P*<0.001) and the no-cage control condition (multiple comparison test with Bonferroni correction, *P<*0.05) (Fig. 3C). The DB condition also showed a marginally significant increase compared with the cage control condition (multiple comparison test with Bonferroni correction, *P<*0.1). There were no significant differences between the NDB and the two control conditions (multiple comparison test with Bonferroni correction, *P>*0.1, remaining pairs).

### Experiment III: ECG measurement for acoustic stimuli

**Fig. 4.**
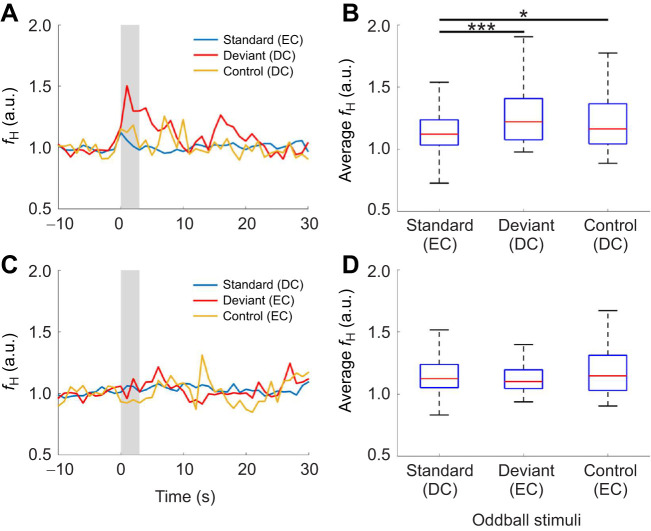
**Results of ECG analysis in experiment III.** The EC-standard paradigm (EC sounds are standard stimuli and DC sounds are deviant stimuli; A,B), and the DC-standard paradigm (DC sounds are standard stimuli and EC sounds are deviant stimuli; C,D). The control session comprised 10 deviant DC stimuli (A,B) or EC stimuli (C,D). Left panels (A,C) show the temporal changes in *f*_H_ of a subject bat from −10 to 30 s. Here, 0 s indicates the onset of stimulation, and the gray area from 0 to 3 s indicates the acoustic stimulation period. Right panels (B,D) show the distribution of average relative *f*_H_ from 0 to 30 s after stimulus onset. Each box plot shows the median (red line), quartiles (bottom and top edges of boxes), and minimum and maximum range (whiskers). Statistical significance is indicated by the asterisks: *P<0.05, ***P<0.001 (multiple comparison test with Bonferroni correction; total of 1255 responses from a total of six subject bats in the EC-standard paradigm; total of 568 responses from a total of four subject bats in the DC-standard paradigm).

We recorded 1255 valid responses from a total of six subject bats in 11 trials using the EC-standard paradigm and 568 valid responses from a total of four subject bats in five DC-standard trials. In the EC-standard paradigm, the deviant DCs caused an increase in *f*_H_ after the onset of the stimuli (Fig. 4A). The averaged *f*_H_ from 0 to 30 s after stimulus onset was significantly different among the three conditions of the EC-standard oddball paradigm (Kruskal–Wallis test, *P*<0.001), but no significant difference was observed in the DC-standard paradigm (Fig. 4C) (Kruskal–Wallis test, *P*>0.1). The *f*_H_ of the deviant stimuli (DC) was significantly higher than that with the standard stimulus (EC) (standard versus deviant, *P*<0.001; standard versus control, *P*<0.05; deviant versus control, *P*>0.1; multiple comparison test with Bonferroni correction) (Fig. 4B). For the DC-standard paradigm, *f*_H_ increases were unclear regarding the stimuli (Fig. 4C). There were no significant differences in *f*_H_ after stimulus onset (standard versus deviant, *P*>0.1; standard versus control, *P*>0.1; deviant versus control, *P*>0.1; multiple comparison test with Bonferroni correction) (Fig. 4D).

## DISCUSSION

### Cognition and a distress situation

In this study, we investigated behavioral and *f*_H_ responses to distress vocalizations in the Japanese house bat *P. abramus*. Using this approach, we tested the hypothesis that an increase in *f*_H_ in subject bats (DC receiver) is caused by the recognition of a distress situation. According to our first result, ‘stay’ was the dominant response in the DB condition, in contrast to the majority of responses involving moving freely in the NDB and control conditions (Fig. 2). In other words, the distress context may have induced an increase in freezing behavior as a fear response in the DC receiver. Freezing behavior has been shown to have led to a rapid increase in *f*_H_ and evoked the dominance of the parasympathetic nervous system in rodents (Liu et al., 2013, 2014; Livermore et al., 2021). The increase in *f*_H_ shown in Figs 3B and 4A may reflect the freezing behavior as a fear response. Hence, our results support the assumption that the freezing behavior was evoked by the recognition of a distress context in a conspecific by the receivers. This suggestion does not conflict with previous physiological studies related to the distress context in bats (Gadziola et al., 2012, 2016; Hechavarría et al., 2020; Ma et al., 2010; Mariappan et al., 2013).

According to a previous study on big brown bats (*Eptesicus fuscus*), *f*_H_ increased by approximately 25% (high aggression) to 50% (lower aggression) during the presentation of 30 s aggressive social calls (Gadziola et al., 2016). In experiment II, *f*_H_ increased by approximately 40% under the DB conditions (Fig. 3B). This response might be a kind of fear response because the *f*_H_ increase was in an analogous range to that in the previous study (Gadziola et al., 2016). The NDB and control conditions may not induce a specific behavioral response, as *f*_H_ was maintained at baseline during these conditions (Fig. 3C).

Considering the possibility that other factors, such as human behavior and olfactory cues, may not have been completely eliminated in experiments I and II, we conducted an additional experiment under conditions of sound presentation with loudspeakers that could completely eliminate these factors. The results of the third experiment (Fig. 4A,B) support the assumption that vocalizations provided distress information to the receiver because *f*_H_ increased by approximately 40% and reached a peak immediately after the onset of deviant DC stimulation during the EC-standard paradigm. Additionally, *f*_H_ gradually returned to baseline within approximately 10 s after the offset of the stimulus. This time course of responses was similar to that of a previous study using aggressive social calls of a few seconds duration in the mustached bat, *Pteronotus parnellii* (Ma et al., 2010). However, DCs did not lead to an increase in *f*_H_ during the DC-standard oddball paradigm, despite using the same stimuli (Fig. 3C,D). In other words, even with the same sound stimulus, the *f*_H_ responses differed depending on the type of deviation. Therefore, there is no doubt that a context consisting of several DCs has a profound effect on the decision making of receiver bats via their autonomic nervous systems. Incidentally, we did not focus on sex or age differences in these responses because the sample size was not large enough to be statistically comparable. Future experiments will examine whether these factors elicit *f*_H_ responses as different internal states. In summary, these results suggest the possibility that *P. abramus* may detect a distress situation from the vocalization context, and that the vocalization context affects the decision making of the receiver bats.

### Vocalization with distress situation in Japanese pipistrelle bats

DCs in Japanese pipistrelle bats, *P. abramus*, had lower TF and/or longer duration than ECs (Fig. 1C), a trend similar to those of other bat species, including *Pipistrellus pipistrellus*, *Molossus molossus* and *Carollia perspicillata* (Carter et al., 2015; Fenton et al., 1976; Gadziola et al., 2012; Hechavarría et al., 2016; Ma et al., 2006; Pfalzer and Kusch, 2003; Russ et al., 1998, 2004). The DCNB was categorized as a high-aggression type according to the categorization of the social calls in previous studies based on the acoustic structure patterns (Gadziola et al., 2012, 2016). In particular, the DCNB had the highest vocalization rate in our experiment. Therefore, our experimental design generally provided a high-distress situation for the subject bats in experiments I and II. In contrast, the DCFM was categorized as a low-aggression type, but this evoked greater *f*_H_ increases than high-aggression calls, such as the DCNB in the receiver bats (Gadziola et al., 2016). In our study, the DCFM, which had the second-largest vocalization ratio in the distress stimulations, increased the relative *f*_H_ by a maximum of 40% in the DB condition, the same as in a previous study (Gadziola et al., 2016). Although we did not compare the *f*_H_ increase with DCNB in our present experiments, the distress context of the DCFM could evoke *f*_H_ increases in *P. abramus*. The difference in *f*_H_ increases between the DCNB and the DCFM may be related to whether the respective vocalizations of social calls are directed toward predators or conspecifics. The DCNB might include mainly aggressive meanings to the predator, despite the DCFM vocalizing with fear escape behavior in our preliminary experiment (Y.N. and K.Y.-H., unpublished data). Therefore, further investigation is needed to determine the possibility of differential effects on physiology and behavior between the DCNB and the DCFM, but it is clear that changes in *f*_H_ are a good indicator for reading changes in internal states induced by the social calls of bats.

The DCs could have multiple roles for conspecifics, predators and other species (Conover, 1994; Klump and Shalter, 2010). In addition, the acoustic characteristics of the DCs in bats may be heterospecific (Arnold et al., 2022; Carter et al., 2015; Chaverri et al., 2018; Russ et al., 2004). In our results, *P. abramus* also showed similar acoustic characteristics to those in other species for the DCs, as shown in Fig. 1A. Therefore, there is a possibility that *P. abramus* could use information from the DCs as intraspecific and heterospecific social calls. Future experiments will test whether these DCs elicit similar *f*_H_ increases in heterospecifics and whether this relies on social relationships between the sender and receiver.

### ECGs of Japanese *Pipistrellus* bats

The ECGs had clear R, S and T waves (Fig. 3A) and were similar to those from previous studies in other bats (Currie, 2018; Mihova and Hechavarría, 2016). The P, Q and U waves were unclear, but this is not a surprising result. The P and U waves could be clarified via averaging as they generally are of small amplitude, normally smaller than the T wave. However, the Q waves of bats remain unclear, as in rodents (Farraj et al., 2011; Kaese and Verheule, 2012; Sambhi and White, 1960). Despite such differences, there are essential similarities between bats, rodents and humans as mammals (Konopelski and Ufnal, 2016). In this study, we focused only on *f*_H_, but other ECG parameters may contain information about the bats' internal state. For example, cardiac arrhythmias are quite interesting, but the details of their relationship with internal states are still unclear and are extremely under-reported in bats (Currie, 2018). In the future, we would like to investigate the relationship between the ECG and the internal state in more detail.

## Supplementary Material

10.1242/jexbio.246271_sup1 Supplementary information

## ECR Spotlight

This article has an associated ECR Spotlight interview with Kazuki Yoshino-Hashizawa.
