# Supplementary material for: The distress context of social calls evokes a fear response in the bat Pipistrellus abramus
Source: J Exp Biol. 2023 Nov 28;226(23):jeb246271. doi: 10.1242/jeb.246271 (PMC10714146; doi:10.1242/jeb.246271)
Supplement: Supplementary information [file jexbio-226-246271-s1.pdf]

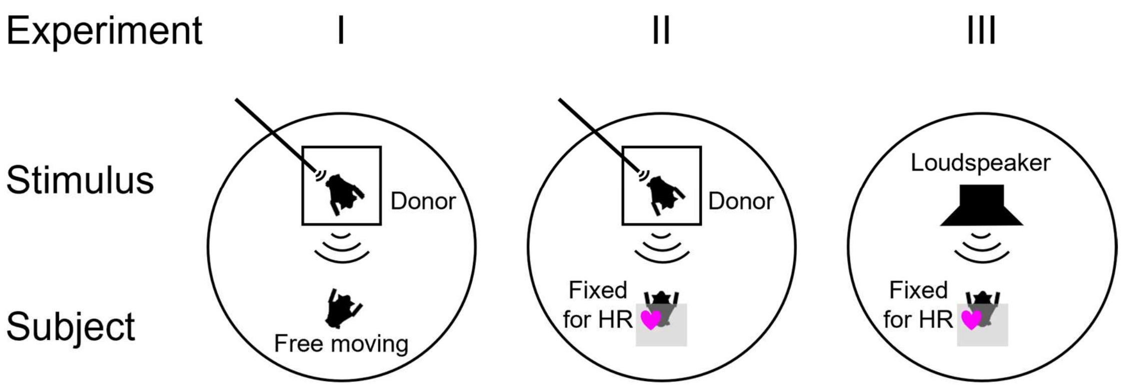

Fig. S1. Abstract scheme of our three experiments.
A donor bat was used as the stimulus in experiments I and II, and a loudspeaker was used in experiment III. In experiment I, the subject bat was allowed to move freely to observe its behavioral response to the donor bat. In experiment II and III, the subject bat was fixed to measure the HR response to each stimulus.

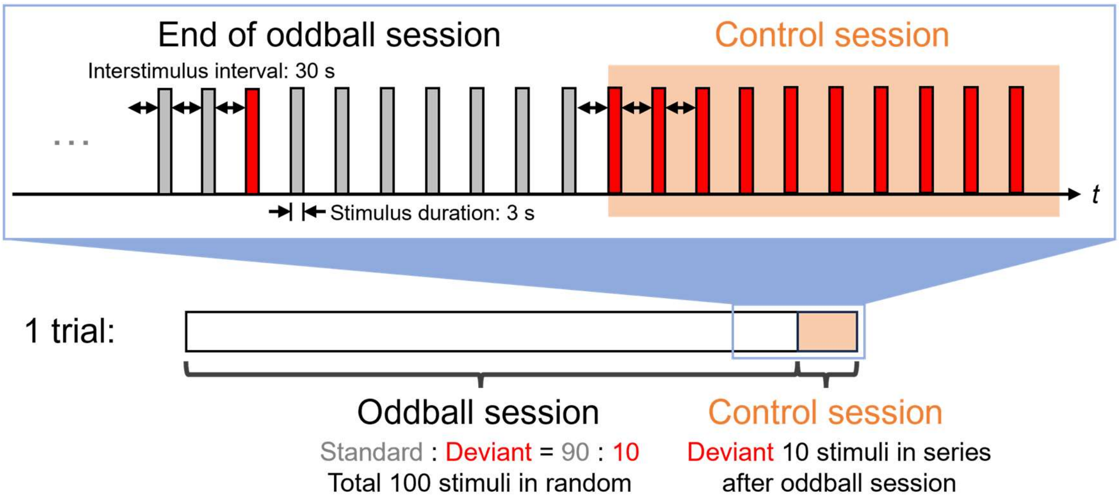

Fig. S2. Scheme of a trial of the auditory oddball paradigm in experiment III.
Each trial consisted of one oddball and one control session as shown in the bottom figure (110 acoustic stimuli in total). First, an oddball session consisting of 90 standard stimuli and 10 deviant stimuli was presented in random order. After 100 stimuli in the oddball session, a control session consisting of 10 deviant stimuli in series was initiated. The figure above shows the details of the presentation near the boundary of the sessions. The gray and red rectangles show the standard and deviant acoustic stimuli, respectively, which were assigned 3 s of EC or DC. All interstimulus intervals were 30 s.

Journal of Experimental Biology • Supplementary information

Table S1. Information from experiment I.

Each trial includes all conditions, except for trial #11, which includes only a control condition. In the DB condition, in addition to the classification of behavioral responses and the reaction time (RT), the number of vocalization calls and the call ratio of each type are listed. Note that the RT is not listed when the response was "stay".

Trial #	Donor	Subject	DB response	DB RT [s]	# of calls	Ratio of EC [%]	Ratio of DCFM [%]	Ratio of DCNB [%]	Ratio of DCO [%]	NDB response	NDB RT [s]	Control response	Control RT [s]
1	M1	F1	Crawl	81	1477	4	19	55	18	Crawl	159	Crawl	168
2	M1	F2	Crawl	165	1138	6	32	38	13	Stay	-	Stay	-
3	M2	F3	Fly	108	971	16	1	59	9	Fly	114	Fly	78
4	M2	F3	Stay	-	1190	0	8	72	8	Fly	114	Fly	33
5	M2	F4	Stay	-	729	13	3	60	8	Stay	-	Crawl	33
6	M1	F4	Stay	-	1532	6	30	50	13	Fly	51	Stay	-
7	M2	F4	Stay	-	1208	1	21	62	10	Fly	162	Fly	60
8	M2	M1	Crawl	27	1041	3	10	75	2	Fly	12	Crawl	18
9	M2	M1	Stay	-	1216	1	14	72	5	Crawl	69	Crawl	39
10	M3	M4	Stay	-	685	24	14	47	5	Stay	-	Fly	84
11	-	M3	-	-	-	-	-	-	-	-	-	Stay	-

Table S2. Recording information details of experiment II.

All pairs were of the same sex to avoid sexual interaction, and four of the pairs were kept in separate rearing cages from the time of capture for the subject bats. One subject-donor pair (M6 and M7) was kept in the same cage from the time of capture. Note that the M6 and M7 pair were tested twice because test #3 was terminated without stimulation of the no-cage control.

Test #	Donor	Subject	Cage	Recording time [s]	Included trials (Total)	DB	NDB	Cage control	No-cage control
1	M4	M5	Separate	1238	13	3	4	2	4
2	M4	M2	Separate	1196	9	2	3	2	2
3	M6	M7	Same	670	6	2	3	1	0
4	M6	M7	Same	646	6	2	2	0	2
5	F5	F7	Separate	849	7	2	3	1	1
6	F6	F7	Separate	1026	13	3	4	2	4
7	F7	F8	Separate	501	6	1	2	1	2

Journal of Experimental Biology • Supplementary information
